# A modified protocol of Capture-C allows affordable and flexible high-resolution promoter interactome analysis

**DOI:** 10.1038/s41598-020-72496-4

**Published:** 2020-09-23

**Authors:** Arkadiy K. Golov, Dmitrii A. Abashkin, Nikolay V. Kondratyev, Sergey V. Razin, Alexey A. Gavrilov, Vera E. Golimbet

**Affiliations:** 1 Mental Health Research Center, Moscow, Russian Federation; 2 Institute of Gene Biology, Russian Academy of Sciences, Moscow, Russian Federation; 3 Faculty of Biology, M.V. Lomonosov Moscow State University, Moscow, Russian Federation

**Keywords:** Chromatin structure, Chromatin analysis, Functional genomics

## Abstract

Large-scale epigenomic projects have mapped hundreds of thousands of potential regulatory sites in the human genome, but only a small proportion of these elements are proximal to transcription start sites. It is believed that the majority of these sequences are remote promoter-activating genomic sites scattered within several hundreds of kilobases from their cognate promoters and referred to as enhancers. It is still unclear what principles, aside from relative closeness in the linear genome, determine which promoter(s) is controlled by a given enhancer; however, this understanding is of great fundamental and clinical relevance. In recent years, C-methods (chromosome conformation capture-based methods) have become a powerful tool for the identification of enhancer–promoter spatial contacts that, in most cases, reflect their functional link. Here, we describe a new hybridisation-based promoter Capture-C protocol that makes use of biotinylated dsDNA probes generated by PCR from a custom pool of long oligonucleotides. The described protocol allows high-resolution promoter interactome description, providing a flexible and cost-effective alternative to the existing promoter Capture-C modifications. Based on the obtained data, we propose several tips on probe design that could potentially improve the results of future experiments.

## Introduction

Spatial proximity between mammalian enhancers and corresponding cognate promoters is thought to be a prerequisite for their regulatory activity. It is widely accepted that the majority of remote regulatory elements come into spatial interactions with promoters of regulated genes^1,2^, although there have been reports that several specific enhancers could distantly activate promoters without direct 3D interactions^3,4^. A hypothesis assuming that genomic enhancers physically approach target genes, which can be located as far as several hundreds of kilobases in the linear genomic sequence, was first proposed in the 1980s^5^; however, compelling experimental evidence of such looping interactions were obtained only in the last 20 years following the introduction of chromosome conformation capture (3C) method in 2002^6^. Based on the proximity ligation principle, which had been originally applied in a more simplistic procedure called nuclear ligation assay^7^, 3C gave birth to a family of methods collectively referred to as C-methods (reviewed in Denker and de Laat^8^).

While there is major variability in promoter–enhancer distance, many enhancers are in fact adjacent to the promoters of their target genes^1,9^. Generally, enhancers are located within the distance of a few tens of thousands of base pairs from the cognate promoters; moreover, it is the closest promoter in the genome sequence that tends to be targeted by the given enhancer. However, this simple rule has many exceptions, and at least some enhancers bypass the nearest genes to activate more distant targets. Furthermore, it is quite common that enhancers regulate transcription of several different genes^10,11^, thus it is impossible to precisely predict regulatory relationships between enhancers and promoters based only on their relative genomic position.

Apart from insight into mammalian transcription regulation processes, description of functional enhancer–promoter landscape appeared to be indispensable for understanding the genetic causes of various common diseases (reviewed in Tak and Farnham^12^ and Nishizaki and Boyle^13^). A large-scale search for the genetic underpinnings of such diseases with GWASs revealed thousands of associated genomic loci. Surprisingly, multiple lines of evidence suggest that causal genetic polymorphisms in these GWAS loci are mostly located within non-coding regions of the genome, presumably in promoters and remote regulatory sequences^14,15^. Hence, prediction of genes that could be regulated by enhancers containing potential disease-causal variants has become one of the key directions of post-GWAS studies, which aim to understand the biological mechanisms of common diseases from the genetic perspective^16–19^.

In recent years, several approaches, both experimental and computational, aimed to better predict links between regulatory elements and specific promoters have been proposed. One of them is based on the estimation of promoter–enhancer spatial proximity using C-methods. It turned out that exceptionally deep sequencing is necessary to reliably detect promoter–enhancer loops in mammalian genomes using completely untargeted, genome-wide C-methods such as Hi-C (all-vs-all methods)^20,21^, which is not yet feasible for most labs. Therefore, promoter–enhancer interactions are commonly assessed with targeted C-methods in which a genome-wide library of proximity ligation products is enriched with ligation products of specific restriction fragments before sequencing^22^. Chronologically first such method, in which PCR is used to enrich 3C library with ligation products pertaining one promoter of interest, was 4C (one-vs-all method): ‘**c**ircular 3C’^23^ or ‘3C-on-**c**hip’^24^. 4C has low scalability. In addition, PCR amplification in 4C is thought to introduce bias, which cannot be accounted for bioinformatically due to the fixed position of read ends on the anchor restriction fragment. The recent introduction of unique molecular identifiers in the 4C protocol addressed the bias problem, but nevertheless, this method is still inconvenient if one wishes to study interactions of more than a few promoters^25^.

A much more scalable hybridisation-based strategy is embodied in promoter Capture-C methods^22^. This group of protocols relies on the in-solution hybridisation of Hi-C or 3C-seq libraries with pools of promoter-targeted biotinylated probes. Library fragments on which biotinylated probes have annealed, are then pulled-down on streptavidin beads, PCR-amplified and sequenced. Post-hybridisation libraries are substantially enriched in the ligation products of probe-targeted promoters with the rest of the genome (many-vs-all methods). Targeted region in Capture-C is readily scaled from several promoters of interest^26^ to genome-wide sets containing thousands of promoters^19,27^. Simultaneously, Capture-C allows the study of the promoter spatial interactome at an exceptionally high resolution currently attainable with Hi-C and other all-versus-all techniques only when they are combined with extremely deep sequencing^20,28^. These features make Capture-C the first choice for data-driven prediction of functional enhancer–promoter pairs.

In all published promoter Capture-C protocols, single-stranded DNA or RNA probes are used for enrichment, making experiments costly or in some cases more labour-intensive^29^. Here, we describe a modification of the promoter Capture-C technique, which is based on 3C-seq library hybridisation with PCR-generated dsDNA probes. We show that such a strategy allows affordable, in-depth interactome profiling for hundreds of promoters in one experiment. We assume that this method could be useful in post-GWAS studies in which the regulatory landscape around GWAS-detected hits is interrogated in relevant cellular contexts.

## Methods

### Probe design and synthesis

**Figure 1:**
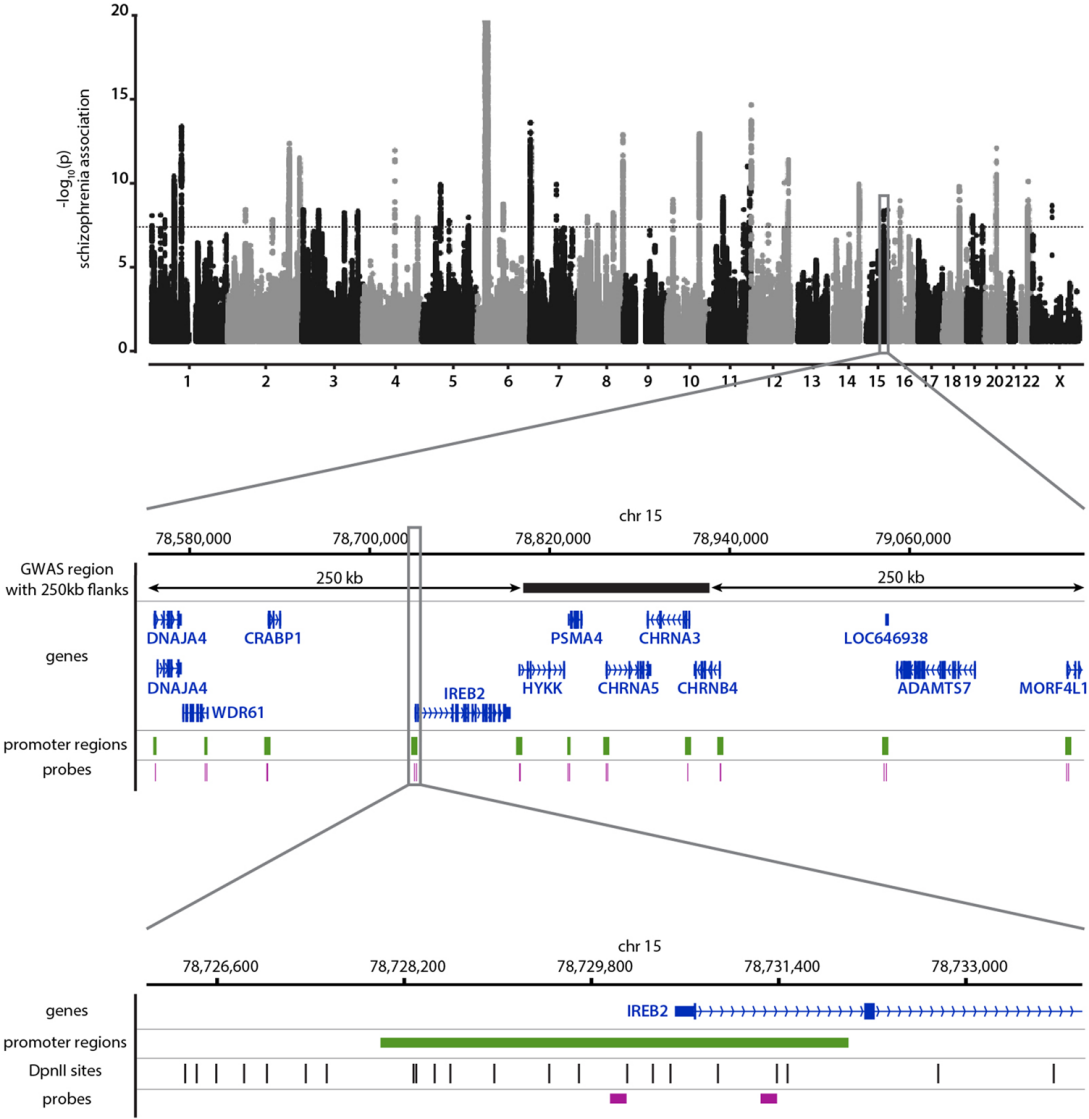
Probes designed for Capture-C experiments. Set of 1651 hybridisation probes was designed for enrichment of 3C-seq libraries with ligation junctions pertaining to 861 promoter regions. Each of these promoters was located within 250 kb from one of 145 schizophrenia-associated genomic regions. Each probe contained 140 bp complementary to one end of DpnII restriction fragments within a promoter region. A Manhattan plot for the latest schizophrenia GWAS meta-analysis^30^ (the graph was plotted with qqman R package based on the GWAS summary statistics downloaded from https://walters.psycm.cf.ac.uk/) and two probes targeting the promoter of *IREB2* are depicted.

A list of promoters was selected based on location inside linkage regions from the latest GWAS for schizophrenia^30^. We excluded 747 genes from the 5 Mbp MHC region on the chromosome 6 because otherwise the list of the target genes would be skewed towards the genes from the single GWAS hit, almost all of which are apparently irrelevant for schizophrenia biology. The borders of the regions were extended by 250 kb from both sides (Fig. 1). Coordinates for the selected genomic regions are listed in Supplementary Table S1. The promoters of genes residing in the regions were selected using the NCBI RefSeq Curated database obtained from the UCSC Table browser. The promoters were defined as TSS (i.e., txStart field for plus strand genes or txEnd field for minus strand) plus 500 bp downstream of a gene and minus 1,000 bp to the other side.

For every promoter the list of DpnII fragments was assigned, ranked by the length of promoter sequence inside the fragment. For every two ends of an untested fragment with a maximum rank, an attempt to find hybridisation probes was made until at least two probes were found to comply with selection rules or until the fragment list was exhausted. The rules were as follows:

1. Each fragment end has only one probe.
2. Probe length is 140 bp.
3. DpnII fragment length is no less than 300 bp.
4. The number of bases in the Repeatmasker base for a probe is no more than 30.
5. The GC content of a probe is no less than 0.25 and no more than 0.65.
6. Each end of the probe is no further than 40 bp to any of the DpnII fragment ends.

With this algorithm, 1651 hybridisation probes were selected for 867 promoters from the original list of 1016 promoters (Supplementary Table S2). The hybridisation probes were flanked by two identical sequences: TCGCGCCCATAACTC on the 5′-end and CTGAGGGTCCGCCTT on the 3′ end. The probes were obtained with a semiconductor-based electrochemical synthesis service, provided by CustomArray, Inc (USA). The sequences of the hybridisation probes are in Supplementary File 1.

For probe preparation, the synthesised pool of oligonucleotides was PCR-amplified with KAPA HiFi HotStart polymerase (Roche, cat.# 07958897001) and 5′-biotinylated forward (5′ bio-CTGGGATCGCGCCCATAACTC 3′) and reverse (5′ bio-CGTGGAAAGGCGGACCCTCAG 3′) primers. As low molecular weight, non-specific PCR products were generated in PCR, we used two rounds of amplification with SPRI bead clean-up in between. Initially, 100 ng of synthetic oligonucleotides was amplified with 19 PCR cycles, then DNA was cleaned up with 1.2 volumes of AMPureXP beads (Beckman Coulter, cat.# A63881) to get rid of low molecular weight, non-specific PCR-products. Six additional PCR cycles were then applied to 20 ng of the purified template, which generated approximately 1 μg of 182 bp dsDNA probes per reaction. Obtained probes were purified with 1.2 × AMPureXP beads (Beckman Coulter, cat.# A63881) and eluted into 10 mM Tris–HCl, pH 8.0.

### Promoter Capture-C

Human HeLa cells were cultured in a DMEM medium supplemented with 10% FBS, 100 U/ml penicillin and 100 U/ml streptomycin at 37 °C in 5% CO_2_ in a humidified atmosphere. Approximately 5 million cells were taken in each experiment. Two biological replicates (HeLa.1.1 and HeLa.1.2) of 3C-seq libraries were prepared according to Golov et al.^31^. A DpnII restriction enzyme was used for chromatin digestion. 2 μg of each post-PCR 3C-seq library were taken in the hybridisation reactions. hybridisation with subsequent washes and PCRs was conducted as described earlier^31^ with the following minor modifications:

1. Per hybridisation reaction, one combined mix (instead of two) was prepared in a PCR tube before DNA melting. Each mix contained 1 ug of sheared salmon DNA (Sigma-Aldrich, cat. #D1626), 10 pmol of Illumina Uni oligo (5′ AATGATACGGCGACCACCGAGATCTACACTCTTTCCCTACACGACGCTCTTCCGATCTC 3′), 10 pmol of Illumina PE 2.0 primer (5′ CAAGCAGAAGACGGCATACGA 3′), 1 μg of 3C-seq library and 500 ng of probes in a total volume of 60 μl.
2. After DNA melting at 95 °C, the hybridisation mix was cooled to 70 °C, then an equal volume of pre-warmed 2 × hybridisation buffer (10 × SSPE buffer, 10 × Denhardt’s solution, 10 mM EDTA, 0.2% SDS) was added to each reaction. Tubes were incubated at 70 °C for an additional 45 min, then the temperature was lowered to 65 °C and hybridisation took place for 20–30 h.

We also made two biological replicates of promoter Capture-C using a modified protocol (referred to as HeLa.2.1 and HeLa.2.2). Several important changes were introduced in this protocol:

1. A 2% formaldehyde solution was used for chromatin fixation instead of 1.5%.
2. Hybridisation reactions were conducted at 60 °C instead of 65 °C.
3. Streptavidin beads were washed with an HS washing buffer (0.1 × SSC buffer, 0.1% SDS) at 55 °C instead of 65 °C.

All four post-capture libraries were subjected to 150 bp paired-end sequencing on an Illumina HiSeq 4000 platform. Between 60 and 85 million pairs of reads were generated for each experiment (Table 1).

**Table 1:** Summary of sequencing and mapping statistics for four conducted promoter Capture-C experiments and for four previously published in situ Hi-C datasets, which were re-analysed in this study.

Experiment type	Promoter Capture-C (current publication)				In situ Hi-C (from Rao et al.^20^)			
Library	HeLa.1.1	HeLa.1.2	HeLa.2.1	HeLa.2.2	HIC084	HIC085	HIC086	HIC087
Number of raw reads	82,936,386	71,316,788	64,412,313	63,915,436	20,968,325	260,998,415	410,229,663	63,173,837
Number of valid pairs (% of total paired reads)	10,024,101 (29.5%)	17,144,141 (39.5%)	6,397,394 (18.3%)	8,341,751 (22.2%)	8,926,592 (42.6%)	124,287,591 (88.9%)	225,136,216 (54.9%)	19,329,884 (52.9%)
Number of unique reads	2,801,403	4,305,870	5,246,239	6,749,255	8,859,531	111,434,931	198,629,485	18,881,718
Number of unique reads falling in baits^a^ (% of unique)	1,930,312 (68.9%)	2,857,913 (66.4%)	2,361,984 (45%)	1,786,248 (26.5%)	47,281 (0.5%)	665,839 (0.6%)	1,216,669 (0.6%)	73,839 (0.4%)

^a^Only 861 promoters targeted with probes in Capture-C experiments were analysed in both Hi-C and Capture-C datasets.

### Capture-C and in situ Hi-C data processing and analysis

Raw Capture-C sequencing reads were mapped on the hg19 reference human genome and filtered using the HiCUP pipeline v0.6.1^32^ (nofill: 1, format: Sanger, di-tag length 100–1000) and Bowtie 2.2.3^33^. For annotation of the promoter interacting regions (PIRs), we used the CHiCAGO R package (version 1.1.8).

CHiCAGO (Capture Hi-C Analysis of Genomic Organisation) is a publicly available pipeline for a robust interaction calling in Capture-C data^34^. CHiCAGO relies on building data-driven two-component convolution background model with distance dependent “Brownian” component and random “technical noise” component. PIRs are identified by comparison of the number of promoter-ligated reads in each genomic bin with the number expected according to the generated model. Final “CHiCAGO score” is assigned to each pair “promoter—genomic bin” by re-weighting of generated *p* values in a procedure which specifically takes into account that larger numbers of tests are performed at promoter–distal genomic regions, where progressively smaller numbers of interactions are expected. Bins with CHiCAGO scores ≥ 5 are considered to be PIRs. The “Brownian” component of the background model in CHiCAGO at least partially accounts for the differences in the local chromatin packing inside nuclei of various cell types as well as for the potential differences in the fixation procedure. Thus, detection of PIRs in CHiCAGO is robust to variations in shape of the scaling plot profiles in different experiments.

BAM files were converted to a CHiCAGO-compatible chinput format with bam2chicago script (https://bitbucket.org/chicagoTeam/chicago/src/master/chicagoTools/). Chinput files are tab-delimited text files that contain basic information about all pairs promoter—“other end” for which at least one ligation product was detected. Each line of the chinput file describes one promoter—“other end” pair, this description includes genomic coordinates of the promoter and the “other end” as well as the number of detected ligation products between this particular pair of bins.

For the CHiCAGO analysis, the reference human genome was split in 2 kb-length non-overlapping bins. Neighbouring bins were merged if each of them was targeted with at least one hybridisation probe. After the merger, we obtained 861 bait bins (or promoter regions) containing all probes, which were roughly equivalent to 867 promoters used during probe design (Supplementary Table S2). Chinput files as well as baitmap and rmap files which were used for their generation are available at the Gene Expression Omnibus under the accession number GSE150048. Total numbers of unique ligation products per each promoter region were computed based on information from the chinput files. We noticed that 21 out of 861 analysed promoters did not have almost any ligation products neither in Capture-C nor in in situ Hi-C HeLa datasets. The majority of these promoters are located in either of two genomic regions on chromosomes 15 (hg19, chr15:82,600,000–83,200,000) and 17 (hg19, chr17:18,925,000–19,125,000), which have low levels of mappability. We excluded 21 aforementioned promoters from further analysis.

For GC-content analysis, only bait bins containing exactly two probes were selected (759 out of 840 remaining promoter regions). The mean GC-content of these two probes were assigned to each of the selected baits as “probe average GC-content” (Supplementary Table S2).

Chinput files from all four conducted experiments were combined for CHiCAGO analyses. For some types of analysis chinput files derived from the original and the modified protocols were submitted to CHiCAGO separately (Supplementary Figure S4). Only non-bait bins with a CHiCAGO score higher than 5 were called PIRs.

In situ Hi-C raw data for four experiments on HeLa cells (HIC084, HIC085, HIC086 and HIC087) were obtained from the database of Genotypes and Phenotypes (dbGaP) through dbGaP accession number phs000640^20^. These datasets were processed identically to promoter Capture-C reads (except usage of nofill: 0 setting in HiCUP).

We used currently unpublished data on topologically associated domain (TAD) boundaries positions in HeLa cells^35^ to analyse localization of detected Capture-C interactions relative to TAD boundaries. To assess the expected number of inter-TAD loops we performed simulations, in which we randomly assigned orientation (forward or backward relative to the reference genome) to the set of intra-chromosomal interactions, which we had described (overall 5107 such interactions). Then we called simulated interactions inter-TAD or intra-TAD depending on whether they crossed a TAD boundary or not. We repeated the simulation 100 times and counted the median number of inter-TAD interactions among all simulations, this figure we called “the expected number of inter-TAD interactions”.

For identification of potentially functional promoter-centred loops in Hi-C and promoter Capture-C data, we intersected genomic regions annotated as enhancers or CTCF-bound sites (in Registry of candidate cis-Regulatory Elements of ENCODE project https://www.encodeproject.org/files/ENCFF280NBC/@@download/ENCFF280NBC.bed.gz) with PIRs from either of the datasets (“bedtools intersect -wao” command in bedtools v2.26.0). We then deleted all duplicated promoter–regulatory element interactions. Lists of discovered loops are available in Supplementary Tables S3 and S4.

For analysis of CTCF motif orientation in loop bases we first selected only those interactions which contain CTCF ChIP-seq peaks (intersection was performed with bedtools v2.26.0) in both promoter and PIR. Then, using HOMER package v.4.11^36^ we searched for CTCF motifs in promoters and PIRs of selected loops (annotatePeaks.pl command). We discarded all loops that do not contain at least one CTCF motif in each of the bases. If a promoter or a PIR contained several motifs we assigned to it a motif with the best score.

To compute PIRs’ enrichment with specific types of regulatory sites and chromatin states, we used the “peakEnrichment4Features” command in the CHiCAGO package. ChIP-seq-predicted positions of four types of regulatory elements (i.e., promoters, enhancers, CTCF-bound sites and other DHSs) in HeLa cells were taken from the Registry of candidate cis-Regulatory Elements of ENCODE project (https://www.encodeproject.org/files/ENCFF280NBC/@@download/ENCFF280NBC.bed.gz). For similar analysis (see Supplemental Fig. S5 and Fig. 4A) we also used chromatin state annotation for HeLa cells generated with chromHMM software within the Roadmap Epigenomics project (https://egg2.wustl.edu/roadmap/data/byFileType/chromhmmSegmentations/ChmmModels/coreMarks/jointModel/final/E117_15_coreMarks_segments.bed). In Fig. 4A, we designated H3K9me3-enriched Het state as “heterochromatin” and H3K27me3-enriched ReprPC and ReprPCWk states as “polycomb-repressed chromatin” for clarity. Location of sequences which show enhancer activity in HeLa S3 cells in STARR-seq experiments were obtained from Muerdter et al.^37^ (https://starklab.org/data/muerdter_boryn_2017/peaks_inhibitor_correctedEnrichment4_supp.table3.tsv). DHS sites were overlaid with STARR-seq peaks using bedtools v2.26.0.

We utilised WashU EpiGenome Browser (https://epigenomegateway.wustl.edu/legacy/) to visualise the detected interactions within the context of relevant functional genomics annotations.

### Downsampling

Raw PCHI-C data were obtained from the PCHI-C Consortium^27^. Sequencing reads pertaining to monocyte, neutrophil and total B cell (TB) ligation products (three replicates for each cell type) were mapped on the hg19 reference human genome and filtered using the HiCUP pipeline v0.6.1^32^ (HindIII, nofill:0, format: Sanger, di-tag length 100–1000). Nine chinput files were generated based on CHiCAGO design files downloaded from https://osf.io/u8tzp/. One thousand baits covered with more than 10,000 unique reads in each of the nine analysed experiments were randomly chosen. Only the rows pertaining to these baits were selected and written in nine modified chinput files. These modified files were analysed using the CHiCAGO R package (version 1.1.8) to generate “full data” sets of PIRs for each cell type. Only non-bait bins falling within a 1 Mb distance from a promoter with a CHiCAGO score higher than 5 were called PIRs. The ligation products from the modified chinput files were then subsampled to obtain chinput files with exactly 10,000 unique ligation products for each bait. These nine once downsampled chinput files were then used as a starting point in 29 rounds of random subsampling. In the first round, 9667 ligation products per promoter were subsampled. In each subsequent round, chinput files with 333 less unique ligation products per promoter than in the previous round were generated. To discover PIRs for each downsampling round, groups of three chinput files for the same cell type and with the same per promoter coverage were combined in the CHiCAGO analysis, which yielded 30 sets of PIRs for each studied cell type. These 90 sets of PIRs and three “full data” sets were overlaid against regulatory data, downloaded from ENCODE. We used three types of regulatory annotations: CTCF-bound sites, enhancers without CTCF and enhancers with CTCF. CTCF-bound sites were retrieved from ChIP-seq data for corresponding cell types (https://www.encodeproject.org/files/ENCFF437LHG/@@download/ENCFF437LHG.bed.gz, https://www.encodeproject.org/files/ENCFF374BNP/@@download/ENCFF374BNP.bed.gz, https://www.encodeproject.org/files/ENCFF449NOT/@@download/ENCFF449NOT.bed.gz), while enhancers were annotated according to cis-Regulatory Elements from the ENCODE project (https://www.encodeproject.org/files/ENCFF348RJV/@@download/ENCFF348RJV.bed.gz, https://www.encodeproject.org/files/ENCFF949VFY/@@download/ENCFF949VFY.bed.gz, https://www.encodeproject.org/files/ENCFF878VCR/@@download/ENCFF878VCR.bed.gz). Enhancers, which are located within 5 kb from the closest CTCF peak, were denoted “enhancers with CTCF” (intersection was performed with bedtools v2.26.0). Promoter–regulatory element pairs conserved between “full data” and 30 k-downsampled datasets were referred to as standard pools of interaction. Finally, we repeated the described analysis for two other sets of 1000 randomly-chosen baits and computed the average retainment of regulatory loops from corresponding standard pools during 29 rounds of downsampling per cell type. Loops with a size more than 0.25 Mb or 0.5 Mb were filtered out from all obtained datasets for additional analysis (Supplementary Fig. S6).

We also downsampled our Capture-C data and data generated by Chesi et al.^38^ in a similar manner. We downloaded raw data generated by Chesi et al. from ArrayExpress (E-MTAB-6862), reads were mapped on the hg19 reference human genome and filtered using the HiCUP pipeline v0.6.1 (DpnII, nofill:1, format: Sanger, di-tag length 100–1000). Chinput files were generated and information pertaining 100 promoters with the highest coverage was selected for downsampling. In this case in the first round data were downsampled to 20,000 unique ligation products per promoter totally (for all three replicates in total). This dataset was stepwise downsampled to 1000 ligation products per promoter. The downsampling procedure was performed with the same set of 100 promoters three times. For PIR regulatory annotation we used CTCF and H3K27ac (as the marker of enhancers) ChIP-seq peaks in primary human osteoblasts from ENCODE (https://www.encodeproject.org/files/ENCFF609ETO/@@download/ENCFF609ETO.bed.gz, https://www.encodeproject.org/files/ENCFF129EHI/@@download/ENCFF129EHI.bed.gz).

For downsampling of HeLa Capture-C data generated in this study we selected 187 promoters with the coverage higher than 15,000 unique ligation products per bait. In this case we started downsampling from 15,000 reads and used for functional loop annotation regions that were called enhancers or CTCF-bound sites in Registry of candidate cis-Regulatory Elements of ENCODE project (https://www.encodeproject.org/files/ENCFF280NBC/@@download/ENCFF280NBC.bed.gz).

## Results

### Biotinylated PCR-products can be used as probes in promoter Capture-C experiments

To enrich 3C-seq or Hi-C libraries with ligation products from specific regions of interest, pools of single stranded RNA or DNA probes are commonly used. Recently we have introduced C-TALE, a novel many-versus-all C-method in which double-stranded 5′-biotinylated DNA fragments generated from BAC DNA are used for targeted enrichment of 3C-seq libraries^31^. BAC clones, which we exploited in C-TALE, harboured long (100–200 kb) inserts corresponding to continuous fragments from genomes of studied species. We wondered whether PCR products targeting discontinuous genomic regions can be used as probes in a similar manner. We were particularly interested in studying promoters, for which several alternative Capture-C enrichment strategies have been developed^22^. To address the feasibility of using dsDNA probes in promoter Capture-C experiments, we purchased a pool of single-stranded synthetic DNA oligonucleotides targeting 861 human promoter regions located inside the genomic regions of schizophrenia GWAS hits or in their immediate vicinity (see “Methods” section). We somewhat arbitrarily chose schizophrenia as an example of a polygenic disease with a large number of potentially relevant genes to validate our protocol. We tried to design a pair of probes for each promoter, but in some cases, we could not pick more than one and in others, merged several closely proximal promoters in one bait region (hereinafter we refer to such merged regions as promoters for simplicity) so that a few of such regions contained more than two probes. Finally, we got 1651 probes, with most promoters (772 out of 861) covered with exactly two probes. Each probe had 140 bases complementary to the end of one of DpnII restriction fragments (Fig. 1); such positioning improves the proportion of valid pairs in sequencing output since we did not use Hi-C-specific pull-down of ligation junctions^26^.

The synthesised pool of 170 base oligonucleotides (140 specific bases plus 15 common bases on either side for primer annealing) was PCR amplified with a pair of 5′-biotinylated primers to obtain dsDNA probes, which were then used in two subsequent hybridisation reactions with 3C-seq libraries generated from HeLa cells. We used HeLa cells to validate our novel experimental strategy as there are plenty of epigenomic data sets for these cells; furthermore, the high-resolution Hi-C^20^ and STARR-seq annotation of sequences with episomal enhancer activity^37^ is also available for this cell line. We used the 4-cutter restriction enzyme DpnII for the preparation of 3C-seq libraries to improve resolution of Capture-C analysis. In addition, we used two rounds of hybridisation to compensate for the inefficiency of dsDNA probes^26,31^. After the final post-hybridisation PCR, libraries were sequenced using the standard Illumina paired end protocol.

**Figure 2:**
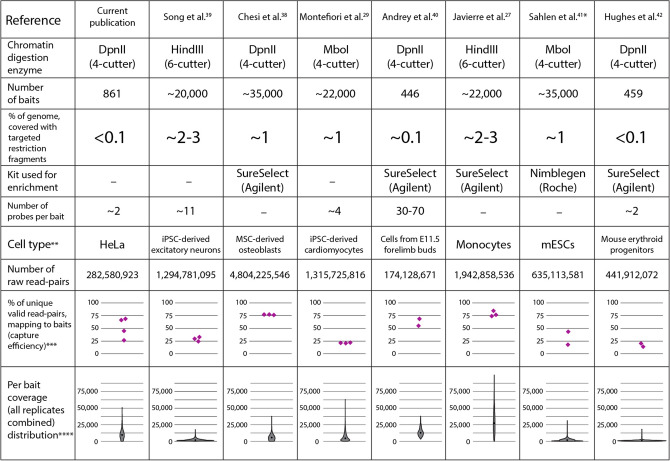
A comparison of the basic features of probe design and principal results of promoter Capture-C experiments in the current publication and several other studies. *All data refers to promoter Capture-C experiments conducted in accordance to HiCap protocol. **In cases where several cell types were studied in a publication, all shown data refers to the indicated cell type. ***All valid read pairs with at least one end mapping to any of the baits are considered as “mapping to baits”. ****Only uniquely mapped reads are considered.

Sequenced reads were mapped to the human genome and then filtered using HiCUP software^32^. Unique valid pairs were afterwards assigned to targeted promoters. A substantial portion (26–66%) of the unique valid read pairs mapped to 861 targeted promoters (Table 1). Although we used a comparatively small set of probes targeting restriction fragments collectively covering less than 0.1% of the genome, two rounds of hybridisation with dsDNA probes gives a similar capture efficiency as conventional protocols of single hybridisation with single-stranded probes (Fig. 2). This data indicates the feasibility of using dsDNA probes for enrichment of 3C-seq and Hi-C libraries.

**Figure 3:**
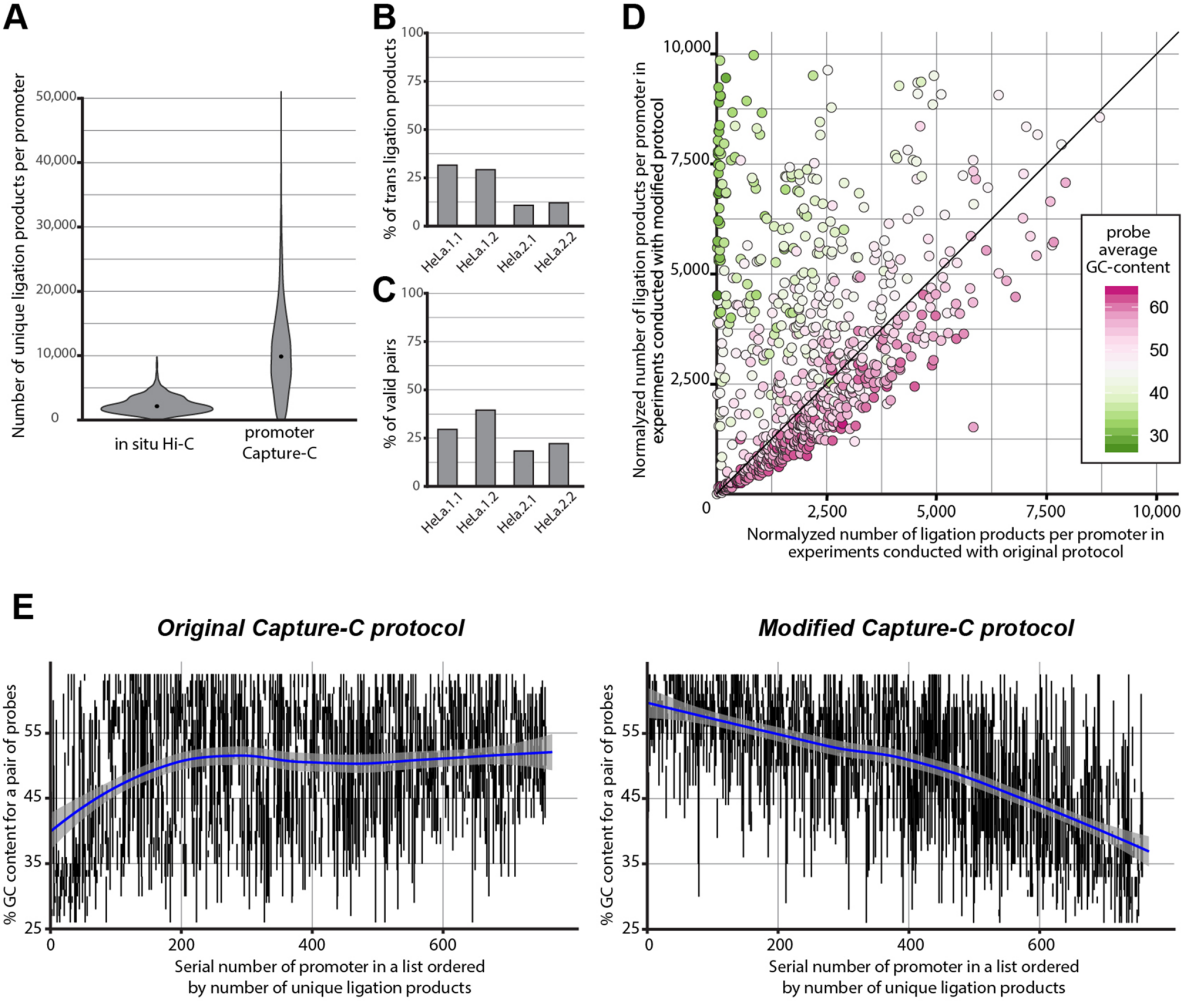
Technical description of the generated Capture-C data. (**A**) Distribution of per promoter coverage for 861 promoters in published Hi-C data for HeLa cells^20^ and in the data obtained throughout the current study. (**B**) Percentage of interchromosomal ligation products among unique valid di-tags in four conducted experiments. (**C**) Percentage of valid di-tags among all aligned paired reads in four conducted experiments. (**D**) Coverage of promoters in experiments conducted according to the modified protocol compared to their coverage in the original protocol. Each point represents one promoter with exactly two probes (promoters with one or more than two probes were discarded, promoters from regions with low mappability are also discarded). Average probe GC-content is colour-coded. To account for differences in restriction enzyme efficiency, coverage was normalised to 10,000,000 unique ligation products. Promoters with coverage of more than 10,000 in either of the experimental conditions are not shown. (**E**) Relationships between probe GC-content and efficiency of oligo capture in experiments conducted in accordance to two different hybridisation protocols (see Supplementary Fig. S1 for the same type of analysis on in situ Hi-C dataset). Each promoter is represented by a vertical line. Only 759 promoters targeted with exactly two probes outside low mappability regions are depicted (see “Methods” section). Position along the y-axis and the length of each line are determined by the GC-content of probes, corresponding to the given promoter. Promoters are depicted according to their coverage in ascending order from left to right. Blue lines with light shadows represent local polynomial regression smoothing models with confidence bands around them.

To further explore the utility of enrichment strategies for detailed analysis of promoter interactions, we compared our data with available high-resolution in situ Hi-C data for the same cell type^20^. For side-by-side comparison, we analysed our data and in situ Hi-C data with the same bioinformatic pipeline focusing on 861 targeted promoters from schizophrenia GWAS regions. Despite a deeper sequencing of Hi-C libraries (approximately three times more total raw reads in Hi-C libraries), it gives substantially worse per promoter coverage than Capture-C (Fig. 3A, Table 1). There is almost two orders of magnitude enrichment in unique reads mapping to targeted promoters in Capture-C libraries.

### Crucial experimental factors influencing quality of promoter Capture-C libraries

In pilot experiments, we performed promoter Capture-C in two biological replicates (HeLa.1.1 and HeLa.1.2) using 1.5% formaldehyde for cell cross-linking. We noticed that the libraries obtained had low cis–trans read ratio, indicating the high proportion of spurious ligation events, which reduce the overall capacity to detect functionally relevant interactions^43,44^. Thus, we raised the concentration of formaldehyde to 2% in our subsequent experiments (HeLa 2.1 and HeLa 2.2). Indeed, this modification increased the cis–trans ratio to a level optimal for C-methods^43,45^ but also led to a decrease in restriction digestion efficiency, which finally manifested in a lower proportion of library fragments containing meaningful ligation junctions (Fig. 3B,C).

We also observed that the majority of valid read pairs in our primary experiments (HeLa.1.1 and HeLa.1.2) are trimmed as PCR-duplicates, which indicates a low complexity of the obtained library and means that the quality of generated data could not be improved by deeper sequencing. This result is potentially due to the use of higher temperatures during enrichment (e.g. for probe hybridisation and streptavidin bead washing) than were originally applied in protocols in which short DNA probes were used for targeted enrichment^41,46^. In addition, we found that many promoters with low coverage in our primary experiments had both their probes with comparatively low GC-content (Fig. 3D,E). This indicated that probes with a low GC-content anneal poorly and can be partially washed from hybridized sequences at 65 °C — the temperature we used for probe annealing and washing out of unhybridised fragments. We therefore decided to make hybridisation and bead washing conditions less harsh to improve the quality of data in the updated variant of the protocol (HeLa 2.1 and HeLa 2.2). Indeed this modification allowed us to acquire slightly better libraries in terms of their complexity. From the same starting amount of 3C ligation product (5 ug), we obtained a similar total quantity of unique reads mapping inside targeted promoters in the improved variant of the protocol (Table 1) despite a nearly two-fold reduction in the number of valid pairs due to decreased restriction efficiency (Table 1, Fig. 3C). On the level of individual promoters we found that coverage of many poorly covered promoters with low GC-content probes was substantially improved (Fig. 3D). This effect was so strong that the correlation between read coverage and average GC-content of probes became inversed compared to the one observed in the primary experimental design (Fig. 3E). In the modified protocol, GC-rich probes on average did not perform as well as probes with a medium or low GC-content. This correlation was also observed in earlier studies^42^ and possibly reflects a propensity of GC-rich sequences to anneal non-specifically during hybridisation. Furthermore, it may indicate that potentially reducing the maximum allowed level of GC-content at the stage of probe design could make the average per promoter coverage in Capture-C experiments higher.

### Enrichment of active chromatin marks at promoter-interacting regions confirms the reliability of the protocol

**Figure 4:**
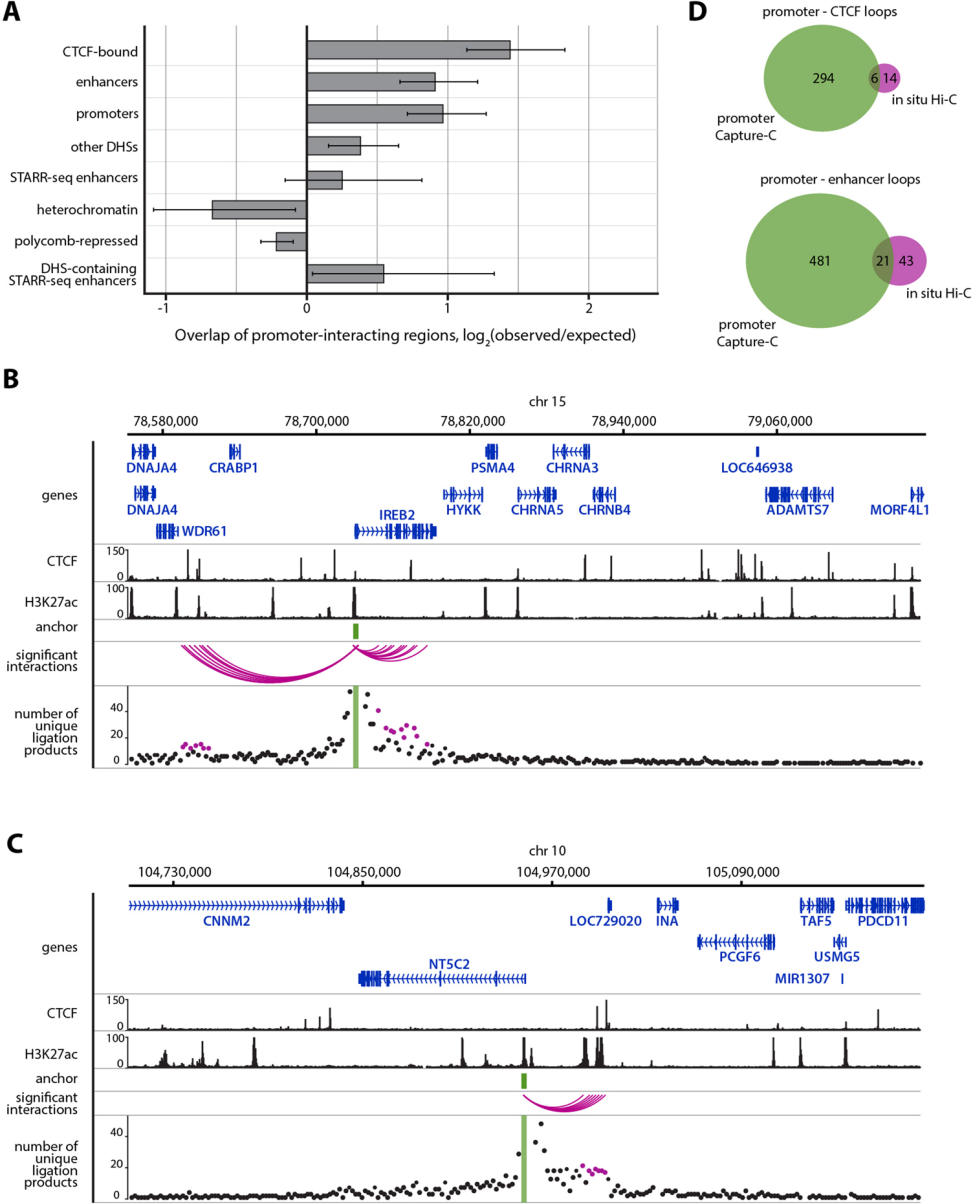
Functional annotation of detected promoter interacting regions (PIRs). (**A**) Analysis of enrichment of various types of genomic sites within PIRs. Enrichment is represented as log_2_ ratio between the observed number of specific types of PIRs and expected number of such PIRs. The latter is computed as average overlap between a random distance-matched set of fragments and corresponding functional annotation. Error bars show s.d. of overlaps across 100 such random sets. (**B**,**C**) Examples of Capture-C-revealed cases of spatial proximity between promoters and H3K27ac-enriched enhancer-like sequences. CHiCAGO-annotated significant interactions represented as arcs between promoters of genes (*IREB2* and *NT5C2*) and PIRs. Points corresponding to the number of unique interactions between promoters and PIRs are depicted in magenta. All represented HeLa ChIP-seq profiles were extracted from ENCODE^47^. (**D**) Promoter–regulatory element loops discovered in the current study and in the previously published in situ Hi-C dataset^20^.

To confirm that the developed protocol is capable of detecting functional promoter interactions on a par with established promoter Capture-C protocols^19,27^, we analysed interaction profiles of 861 studied promoters with the CHiCAGO R package, which is designed to identify promoter-interacting genomic regions (PIRs) in promoter Capture-C data^34^. When combining data from all four conducted experiments, we were able to identify 5,241 PIRs, the vast majority of which resided within the same topologically associated domain (TAD) as the corresponding anchor promoter (Supplementary Figure S2). For further analysis we selected 5,039 PIRs located closer than 1 Mb from their anchors. These PIRs were significantly enriched with CTCF-bound sites as well as active enhancers and promoters which were annotated according to the presence of specific active histone modifications (Fig. 4A–C). Overall, 300 unique promoter–CTCF loops and 502 promoter–enhancer loops were identified (Supplementary Tables S3 and S4). Interestingly, only a small portion of these loops was detected when in situ Hi-C data from the same cell type were re-analysed in the same way, which highlights the importance of high level of per promoter coverage when studying regulatory spatial chromatin interactions (Fig. 4D). Some loops, however, are exclusively detected in Hi-C data. In a few cases such Hi-C specific loops reflect the fact that some baits in Capture-C data have extremely low coverage. In other, more prevalent cases, these Hi-C specific loops, when visually inspected, turn out to be individual outlier bins with a few ligation products, which indicates their stochastic, likely false positive nature. On the other hand loops detected in both datasets tend to be represented in Hi-C data as clustered groups of significant bins (PIRs) in comparatively well-covered regions (Supplementary Figure S3).

In our Capture-C data we also detected 65 loops, which have CTCF ChIP-seq peaks and motifs for CTCF binding on both anchors of the loop. In agreement with previous publications^20,48^ convergent motif orientation was significantly overrepresented among such loops: 42 out of 65 CTCF motif pairs have convergent orientation (p = 1.906*10^-11, binomial test).

To further confirm superiority of our modified protocol in comparison with the original one we annotated promoter–enhancer and promoter–CTCF loops detected in data from either of the protocols in separate analysis. Despite slightly lower median per promoter coverage in data obtained with the modified protocol (4,037 reads per promoter vs 5,067.5 in the original protocol, see Supplementary Figure S4), analysis of these data yields higher proportion of functional loops. 78.3% (235 out of 300) of promoter–CTCF loops and 74.3% (373 out of 502) of promoter–enhancer loops described in the analysis of merged dataset can be also detected in data from the modified protocol, whereas only 55.7% (167) and 68.5% (344) of respective loop types can be detected in data derived from the original protocol (Supplementary Figure S4, Supplementary Tables S3 and S4).

The results of STARR-seq experiments cataloguing genomic fragments potentially capable of enhancing transcriptional activity in HeLa S3 cells have been recently published^37^. To further assess the ability of Capture-C to detect functional promoter–enhancer loops, we compared the location of detected PIRs with the location of STARR-seq peaks. Surprisingly, we have not discovered significant overrepresentation of the STARR-seq signal in PIRs (Fig. 4A). One possible explanation for this is that the STARR-seq signal is based on the enhancer activity of sequences in episomal constructs, outside of their native epigenomic context. In fact, authors of the STARR-seq publication noticed that almost half of the discovered STARR-seq peaks resided in heterochromatic regions and therefore possibly did not exhibit enhancer activity in situ. On the other hand, it is known that PIRs are usually depleted of heterochromatin-specific histone modifications, such as H3K27me3 and H3K9me3^19,27^. We actually observed depletion of at least some types of repressed chromatin in PIRs in our data (Fig. 4A, Supplementary Fig. S5). We then hypothesised that since both functionally active enhancers and potentially active sequences repressed by heterochromatin are present among STARR-seq peaks in substantial proportions, we cannot detect neither enrichment nor depletion of the STARR-seq signal in PIRs. Indeed, those STARR-seq peaks which contain DHS sites are overrepresented in Capture-C PIRs (Fig. 4A).

### Assessing the per promoter read coverage sufficient for comprehensive annotation of promoter spatial interactions

**Figure 5:**
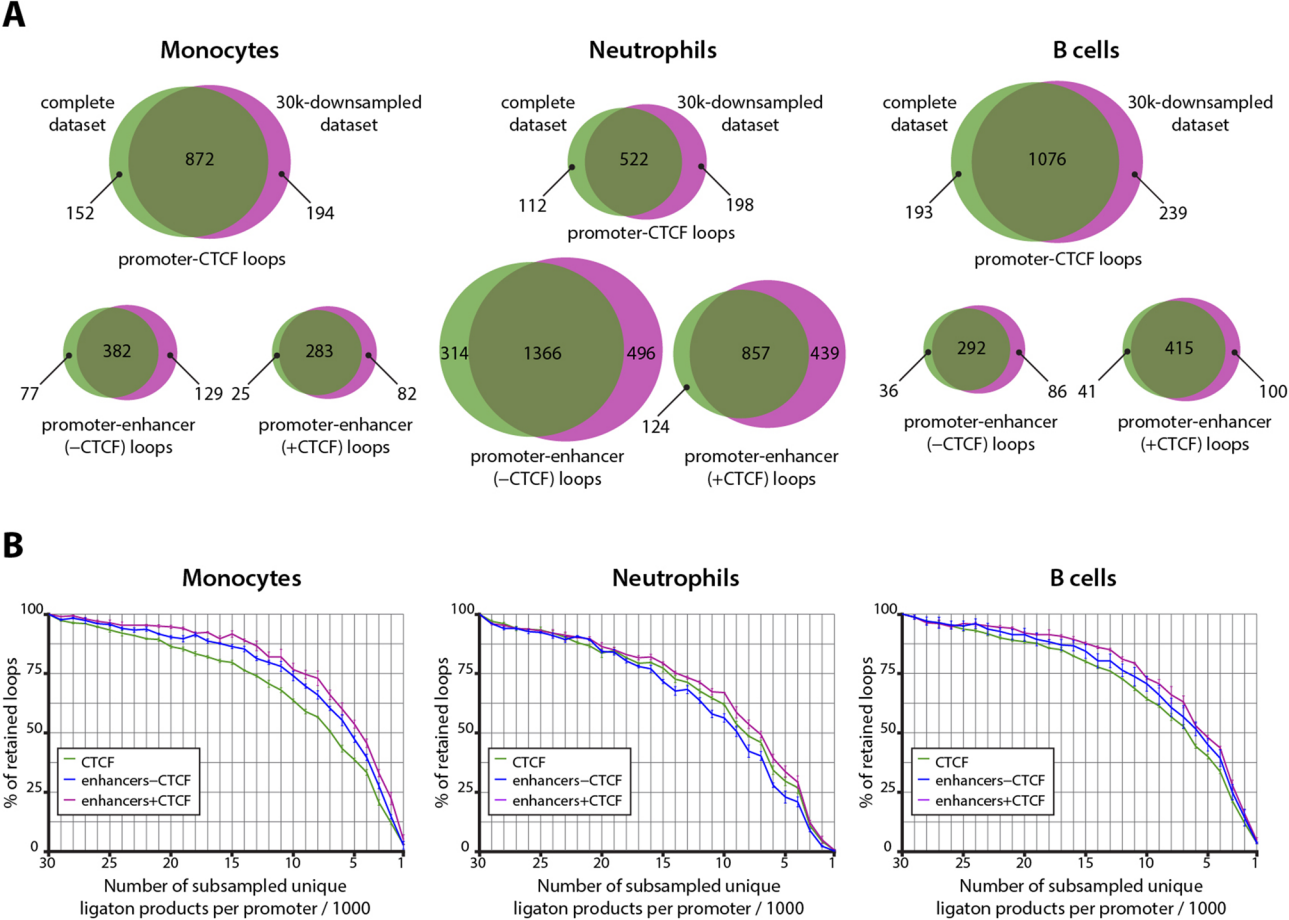
Retention of functional loops being detected during promoter Capture-C data downsampling. (**A**) Venn diagrams, which show CHiCAGO-detected functional loops pertaining to 1000 randomly chosen promoters, in complete datasets (green circles, median numbers of ligation products per promoter are 50,251 for monocytes, 64,827 for neutrophils and 63,743 for B cells) and subsampled datasets (magenta circles), containing exactly 30,000 ligation products per promoter. All diagrams show one representative downsampling experiment for each cell type; data on the remaining six experiments in Supplementary Fig. S6. Note substantial number of apparent false positive loops arising in the process of subsampling. (**B**) Proportion of CHiCAGO-detectable loops retained in datasets subsampled to different levels of per promoter coverage. Loops conservatively detected in both complete and 30 k-downsampled datasets were considered to comprise the standard pool of interactions (100%). Different classes of PIRs are depicted in different colours. Averages of three independent downsampling experiments are shown, error bars represent s.d.

In terms of per promoter read coverage, we note that the quality of promoter Capture-C data varies drastically among different publications (Fig. 2). For example, some promoter Capture-C experiments have a median of around a few thousand unique reads per promoter^39,41,42^, whereas in others, most promoters have over 10,000 unique ligation products^27,40^. Moreover, different promoters in one experimental setting are covered unequally. In our HeLa data, we observed that some promoters are poorly covered (i.e., 107 promoters have less than 2,500 unique ligation products), whereas coverage of others is much higher (i.e., 187 promoters have over 15,000 unique ligation products). Simultaneously, comparison between Capture-C results and Hi-C results suggests that coverage could significantly influence completeness of promoter-centred interaction annotation (Fig. 4B). Thus, we wondered whether there is some value at which further increases in per promoter coverage cannot drastically improve annotation of spatial promoter interactions. To address this problem, we analysed one of the most well-powered promoter Capture-C datasets obtained for 17 types of human primary blood cells^27^. We selected three cell types: monocytes, total B cells and neutrophils, and all promoters that have over 30,000 unique ligation products total in all three cell types. Next, we randomly took 1,000 of such promoters and, with the help of the CHiCAGO package, detected significant interactions these promoters establish with different types of regulatory elements active in the corresponding cell types (see “Methods” section). Afterwards, we randomly downsampled per promoter coverage in each cell type to 30,000 and reanalysed these truncated datasets with CHiCAGO. Surprisingly, we found that not only some fraction of interactions is lost during this first round of downsampling, but that a comparable number is gained despite reduction in coverage (Fig. 5A). This suggests that at least some CHiCAGO-detected promoter interactions are false positives. To decrease contribution of these sporadic interactions in the subsequent analysis, we took only interactions detected in both the whole dataset and the 30,000-downsampled (30 k) dataset as a standard pool of interactions. We then used 30 k datasets as a starting point for 29 rounds of random sampling. Each sampling contained 1,000 less ligation products per promoter than the previous one thus we ultimately acquired 30 downsampled datasets for each cell type containing 1,000; 2,000; 3,000 etc. interactions per each of the 1,000 chosen promoters. Next, these subsampled datasets were analysed with CHiCAGO and the detected promoter-centred interactions were compared to the standard pool of interactions for each cell type. The described procedure was repeated for two other random sets of 1,000 promoters to get three replicates of downsampling for each cell type. We then plotted the percentage of retained interactions against a number of per promoter ligation products in each cell type (Fig. 5B). The steady but very slow decrease in the number of detected interactions was observed until number of ligation products per promoter achieved 15 k. One could speculate that a substantial portion of interactions lost during this stage are actually false positives, accidentally detected in both the whole and 30 k datasets. When the number of ligation products falls below 10 k, the number of retained CHiCAGO-detectable contacts begins to decline dramatically. Anyway, for all studied types of contacts in all cell types, it is true that a very high proportion of potentially functionally significant loops (i.e., approximately 60% of promoter–CTCF loops and 55–75% of enhancer–promoter loops at distances below 1 Mb, and more than 70% of any type of interactions at distances below 250 kb) could be detected with CHiCAGO in datasets with coverage of 10,000 ligation products per promoter (Fig. 5B, Supplementary Fig. S6).

**Figure 6:**
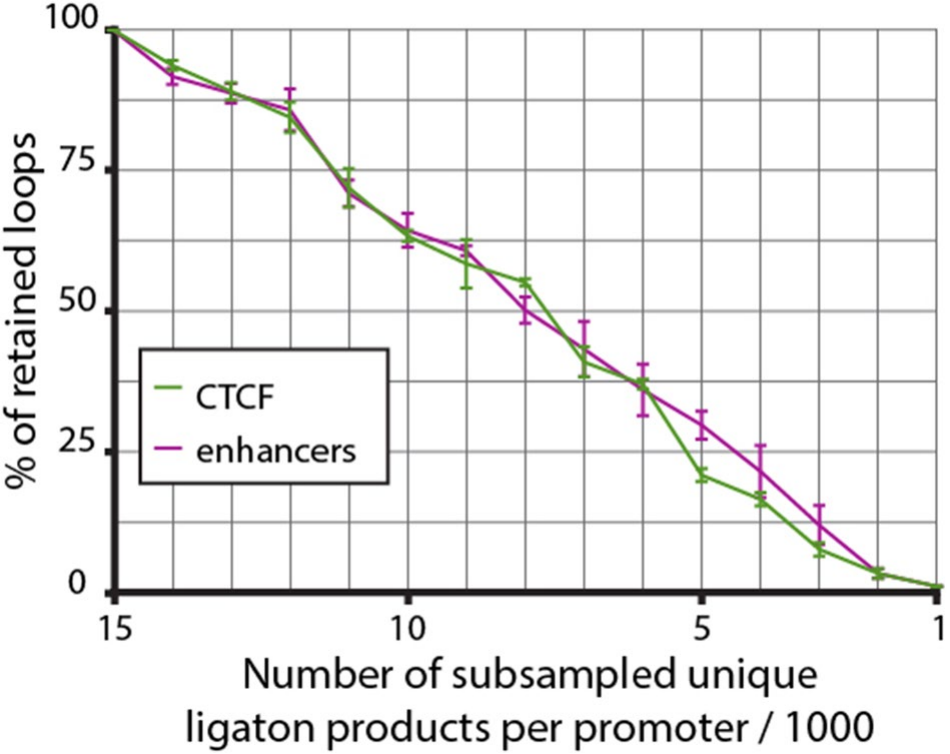
Retention of functional loops being detected during downsampling of promoter Capture-C data generated in this study. Only 187 best covered promoters were used for the analysis. Loops conservatively detected in both complete and 15 k-downsampled datasets were considered to comprise the standard pool of interactions (100%). Different classes of PIRs are depicted in different colours. Averages of three independent downsampling experiments are shown, error bars represent s.d.

To test whether the described dependency between per promoter coverage and the ability of the C-method to detect promoter-centred loops is valid for other experimental settings, we performed similar downsampling analysis for 100 best-covered promoters from Chesi et al.^38^ (Supplementary Figure S7) and 187 promoters with > 15,000 reads per promoter in our HeLa dataset (Fig. 6). In both cases we observed a dramatic decrease in fraction of detectable loops over the course of downsampling, with less than a third surviving downsampling below 5,000 reads per promoter. We noticed specifically that only a few percent of loops in HeLa dataset remain detectable at 2,000 reads per promoter coverage (median coverage of in situ Hi-C dataset, see Fig. 3A), which further supports our notion that low coverage is the main reason for low number of CHiCAGO-detectable loops in Hi-C data (Fig. 4B).

Overall, we assume that the number of 10,000 ligation products per promoter can be considered a benchmark for promoter Capture-C experiments.

Application of this quality standard to our data shows that only half of the studied promoters are sufficiently well-covered (414 of 861 have coverage more than 10,000), meaning that we seemingly cannot detect a significant fraction of loops for the other half. We believe that, apart from increasing the number of replicates and depth of sequencing, the quality of obtained data could be improved by modifications in probe design, some of which will be described below.

## Discussion

Single-stranded RNA or DNA probes are commonly used to enrich 3C-seq and Hi-C libraries with specific types of ligation products. The double-stranded probes are thought to be not as efficient as single stranded ones since they tend to reanneal on each other during hybridisation. On the other hand, the usage of PCR to amplify synthetic DNA oligo-precursors of probes is very convenient since this allows the generation of vast amounts of probes for many hybridisation reactions from oligos purchased once. Some protocols have already taken advantage of this and exploited PCR to generate DNA templates for subsequent in vitro transcription, which produce biotinylated ssRNA probes for targeted enrichment of Hi-C libraries^29,49^. Meanwhile, we noticed that some researchers have already used dsDNA to enrich sequencing libraries in fragments from regions of interest^50^ and recently introduced C-TALE—novel hybridisation-based many-versus-all C-method in which dsDNA probes are exploited^31^. We showed that the comparative inefficiency of dsDNA probes could be compensated with an additional round of hybridisation without introducing significant biases in final contact maps.

Synthesis of custom pools of long oligonucleotides is considerably cheaper than purchase of commercial kits for targeted enrichment. Thus PCR-generated dsDNA probes make Capture-C experiments less expensive, especially in those cases when the study is focused on a small set of promoters (in the range of several hundreds) since in such experiments the fraction of expenditures associated with sequencing is comparatively small and the price of probes becomes the crucial factor. In projects where interactions of the same set of promoters should be analysed in various different cellular models the usage of PCR-generated dsDNS probes seems even more beneficial because, in contrast to probes from the commercial kits, they can be generated in almost unlimited amounts from precursors synthetised once.

We show that dsDNA probes generated from a pool of long synthetic oligonucleotides could be successfully used in promoter Capture-C experiments, resulting in high resolution maps of spatial interactions for sets of several hundred promoters. This version of the probe preparation protocol is reasonably simple and makes Capture-C experiments more affordable and flexible. Computational analysis of the obtained data showed that the number of promoter-centred interactions detected under described Capture-C protocol is comparable with numbers reported for other variants of the Capture-C (on average ~ 6 interactions per promoter in our data, while 4–7.5 interactions were described in previously published studies^27,34^). Moreover, enrichments of CTCF-binding sites and enhancer-specific chromatin marks in the PIRs (~ 2.7X for CTCF and ~ 1.88X for enhancers), which we detected using our new protocol, were similar to the enrichments described in the finest specimens of Capture-C data published to date (2–3X enrichment)^19,27^. Overall, we conclude that the described version of Capture-C is able to detect promoter–enhancer and promoter–CTCF loops on a par with pre-existing variants of the Capture-C procedure. Our protocol may be especially useful in experiments aiming to study regulatory interactions in and around genomic regions annotated as genetically associated with specific traits by GWASs. We hope the Capture-C protocol described here will be included in the toolbox of methods available for in-depth study of promoter-centred spatial interactions.

We have also studied the effect of per promoter read coverage in Capture-C on capability to detect spatial interactions between promoters and various types of remote regulatory elements. We discovered that the higher per promoter coverage results in an improved ability to detect promoter-centred loops, which was to be expected. However, we argue that certain coverage, namely 10,000–15,000 unique read pairs, allows one to detect the most part of the functionally relevant spatial interactions of a given promoter. We have found that even comparatively high-resolution Hi-C data are significantly underpowered for the deep analysis of promoter interactome. Superiority of Capture-C data in terms of coverage for targeted promoters is something that could be expected beforehand given substantially higher complexity of Hi-C libraries (Fig. 3A). However, we found that this low coverage of individual promoters in high-quality Hi-C data leads to incapability to discover most promoter–enhancer and promoter–CTCF loops (Fig. 4B).

We recognise that coverage of approximately half of the promoters (447 out of 861) in our dataset do not comply with the above-mentioned threshold value, and we are unable to detect some interactions of these genes. Furthermore, comparatively low coverage of many promoters in our data prompted us to analyse promoter interactions in 2 kb genomic bins. It is believed that such binning can potentially introduce biases associated with uneven distribution of restriction sites among different bins^51^. These biases could additionally hinder our ability to detect promoter loops or introduce false positive ones.

In our experience, several factors make major contributions to the final coverage of a given promoter in Capture-C analysis, the most obvious being the sequencing depth. Although at some point, one cannot improve coverage with further sequencing as the complexity of the final Capture-C library becomes a limiting factor. This complexity is known to be partially determined by the complexity of the initial 3C-seq (Hi-C) library. As Hi-C libraries prepared from the same amount of starting materials are typically much less complex than 3C-seq libraries (due to inefficiency of proximity ligation in Hi-C), we usually opt to work with 3C-seq protocol, even though we eventually obtain a much worse proportion of valid pairs in sequencing output. Complexity of the initial library and choice of 3C-seq protocol could become crucial when dealing with small numbers of cells (less than 1 mln). Another major contributor to the complexity of Capture-C libraries is enrichment procedure itself. Due to inefficiency of the hybridisation reaction the most part of the targeted restriction fragments do not end up binding to the streptavidin beads. Hybridisation with double-stranded probes is seemingly less effective; therefore, we started with a large amount of 3C-seq library (several micrograms) in the first round of enrichment. Besides, we observed that harsh washing conditions can substantially decrease the overall complexity of Capture-C libraries in case of exploitation of short DNA probes and opted to use milder washing conditions. Finally, per promoter coverage in Capture-C is heavily influenced by probe design, specifically the number of probes per promoter, probe GC-content and probe sequence. Differences in the capability of probes to capture targeted restriction fragments enhances the disparity in per promoter coverage in Capture-C data. Like other authors^42^, we noticed that probes with a high GC-content generally perform worse, at least in the hybridisation conditions we ultimately used; however, there are multiple properties of each specific sequence, which we cannot account for and which can potentially lead to poor performance of the probe. For this reason, several probes are usually designed for each promoter in Capture-C as a fail-safe measure, and in all likelihood, more probes will give better and more uniform per promoter coverage^40^.

We believe that the shortcomings of probe design in our current experimental settings are major reasons leading to unsatisfactory coverage of substantial part of promoters. Several potential improvements could be introduced to mitigate these shortcomings. Based on obtained data, we suppose that per promoter coverage could benefit from decreasing probe GC-content since GC-rich probes perform worse under the described experimental conditions. Another reasonable optimisation strategy is increasing the number of probes per targeted promoter. We used two probes per promoter, but the usage of a 4-cutter restriction enzyme at the 3C stage makes it possible to engage significantly more restriction fragment ends for each promoter during the probe design^40^. We anticipate that designing four to ten probes per each promoter for Capture-C experiments on promoter sets of such size (i.e., several hundreds) will significantly improve coverage level and uniformity while only slightly changing overall expenditures.

## Supplementary information

Supplementary Figures.

Supplementary Information.

Supplementary Tables.

## Data Availability

Raw Capture-C sequencing reads generated in this study were deposited to the database of Genotypes and Phenotypes (dbGaP), as a substudy under accession number phs000640. CHiCAGO design files as well as chinput files for 8 analysed HeLa sequencing libraries are available at the Gene Expression Omnibus under the accession number GSE150048.
