# Supplementary material for: A modified protocol of Capture-C allows affordable and flexible high-resolution promoter interactome analysis
Source: Sci Rep. 2020 Sep 23;10:15491. doi: 10.1038/s41598-020-72496-4 (PMC7511934; doi:10.1038/s41598-020-72496-4)
Supplement: Supplementary file 1 — Supplementary Figures. [file 41598_2020_72496_MOESM1_ESM.pdf]

A modified protocol of Capture-C allows affordable and flexible high-resolution promoter interactome analysis

Arkadiy K. Golov*[1,2], Dmitrii A. Abashkin[1], Nikolay V. Kondratyev[1], Sergey V. Razin[2,3], Alexey A. Gavrilov[2], Vera E. Golimbet[1]

[1] Mental Health Research Center, Moscow, Russian Federation
[2] Institute of Gene Biology, Russian Academy of Sciences, Moscow, Russian Federation
[3] Faculty of Biology, M.V. Lomonosov Moscow State University, Moscow, Russian Federation
* Corresponding author (E-mail: golovstein@gmail.com)

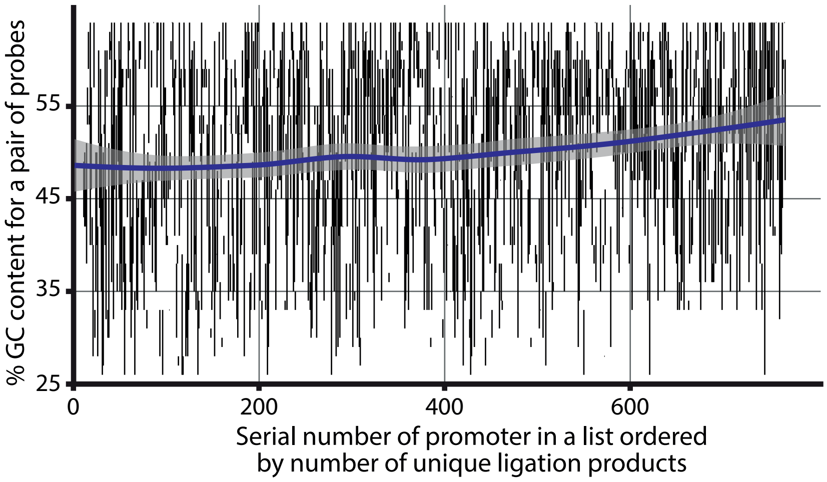

Figure S1. Relationship between GC-content of probes used in the current study and per promoter coverage in previously published Hi-C data[20]. Each promoter is represented by a vertical line. Only 759 promoters targeted with exactly two probes outside low mappability regions are depicted (see "Methods" section). Position along the y-axis and the length of each line are determined by the GC-content of probes, corresponding to the given promoter. Promoters are depicted according to their coverage in ascending order from left to right. Blue line with light shadow represents local polynomial regression smoothing model with a confidence band around it.

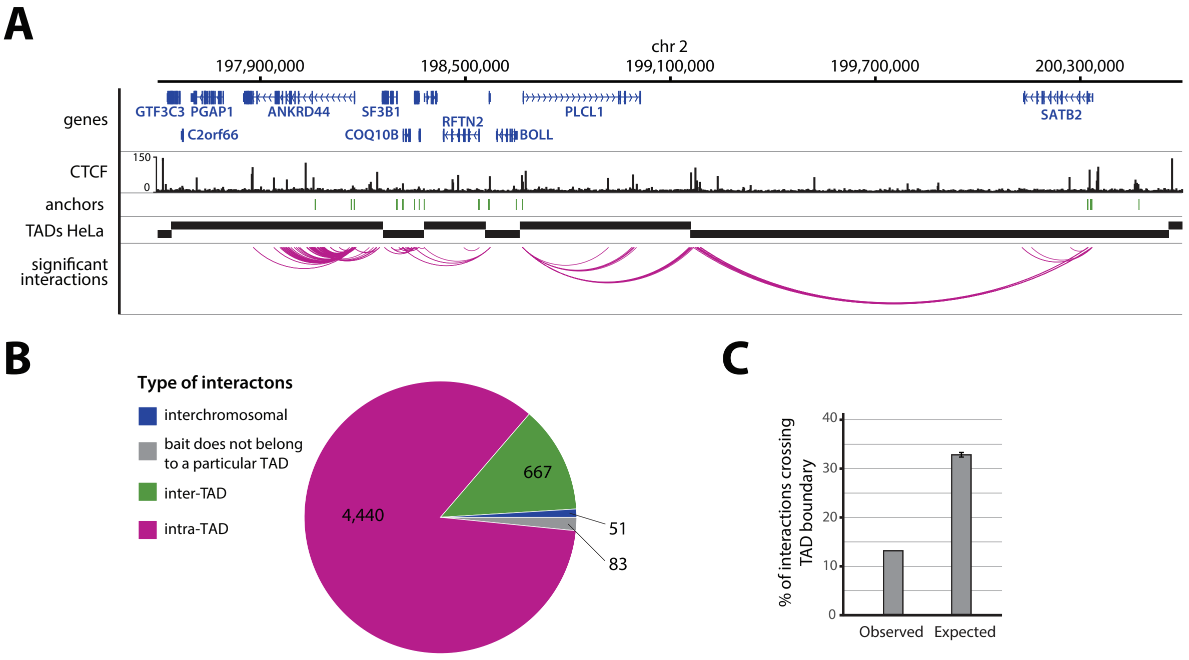

Figure S2. Both anchors of Capture-C interactions tend to reside within the same topologically associated domains (TADs). A. An example of an extended region analyzed in the current study. Note that the majority of promoter-centred interactions do not cross the TAD boundaries. B. Classification of detected Capture-C interactions based on their localization relative to the TAD boundaries. C. Proportion of interactions crossing TAD boundaries, error bars represent s.d.

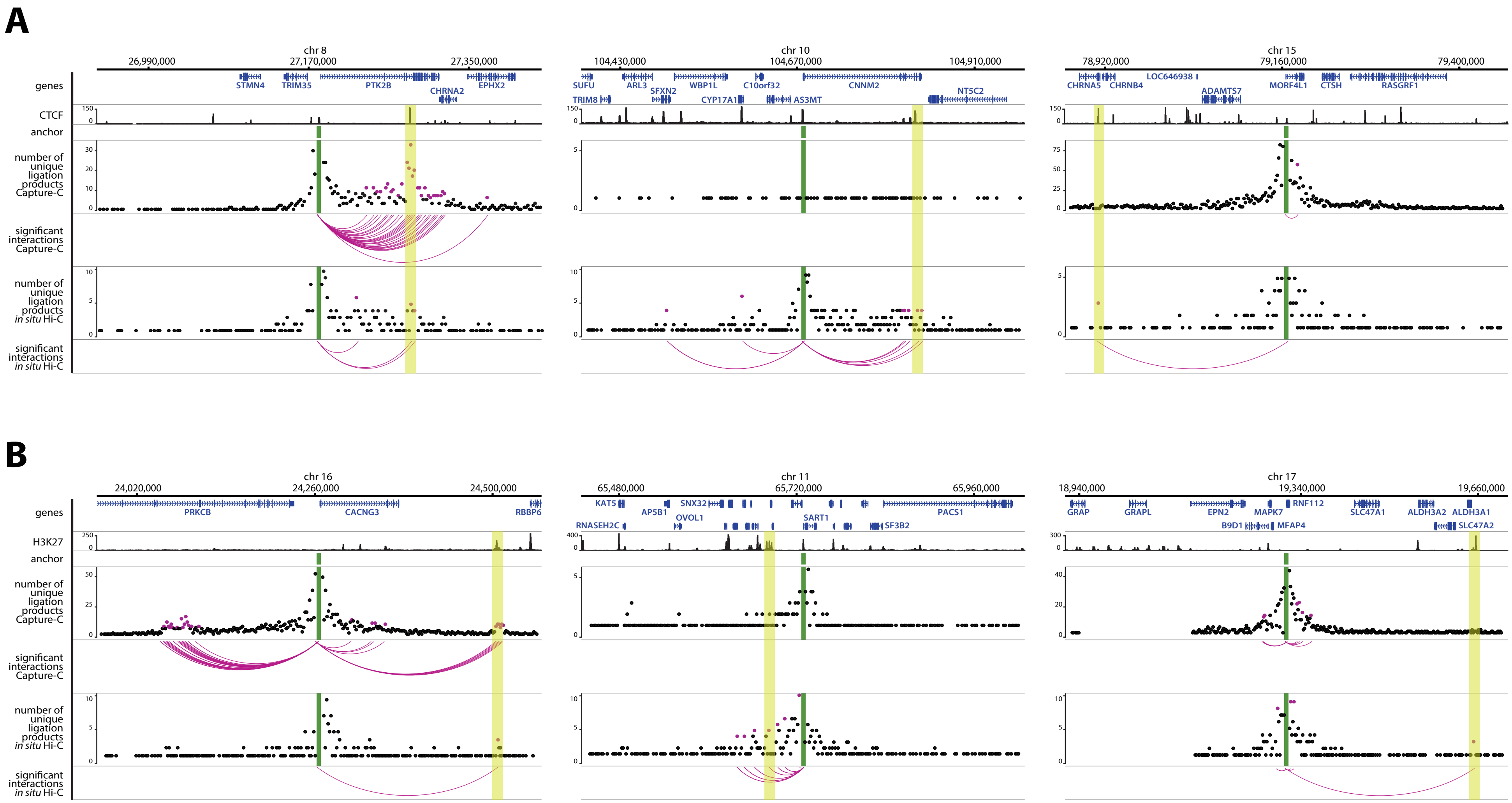

Figure S3. Functional loops detected in both Capture-C and in situ Hi-C datasets, and loops observed specifically in *in situ* Hi-C data. Points corresponding to the number of unique interactions between promoters and PIRs are depicted in magenta. Yellow bar in each panel highlights the position of PIR which we specifically focus on. All represented HeLa ChIP-seq profiles were extracted from ENCODE[47]. A. Promoter–CTCF loops. Left panel shows a loop common for both datasets, whereas the central and the right panels depict Hi-C-specific loops. Note that the central one is not detected in Capture-C data due to an extremely low coverage, whereas the right one represents an example of false positive Hi-C-specific loop. B. Promoter–enhancer loops. Left panel shows a loop common for both datasets, whereas the central and the right panels depict Hi-C-specific loops. Note that the central one is not detected in Capture-C data due to an extremely low coverage, whereas the right one represents an example of false positive Hi-C-specific loop.

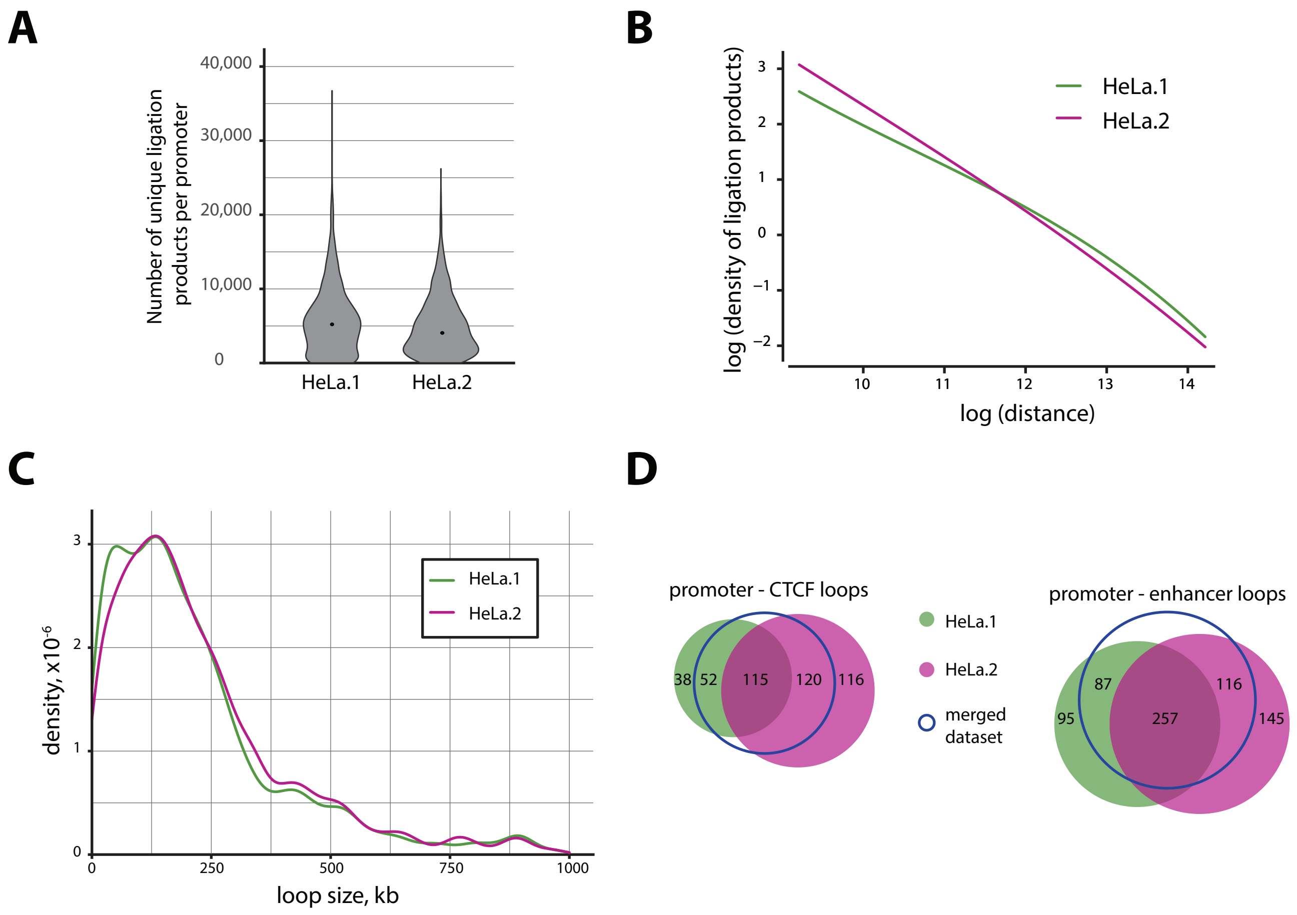

Figure S4. Separate analysis of Capture-C data obtained with the original (HeLa.1) and the modified (HeLa.2) protocols. A. Distribution of per promoter coverage for 861 studied promoters. B. Double logarithmic scaling plot of the dependence of contact probability on genomic distance. C. Distance dependency of significant interaction density. D. Promoter-regulatory element loops discovered in the merged dataset and loops which can be detected when the data from the original and the modified protocols are analyzed separately. Note that there are totally 300 promoter–CTCF loops and 502 promoter–enhancer loops in the merged dataset.

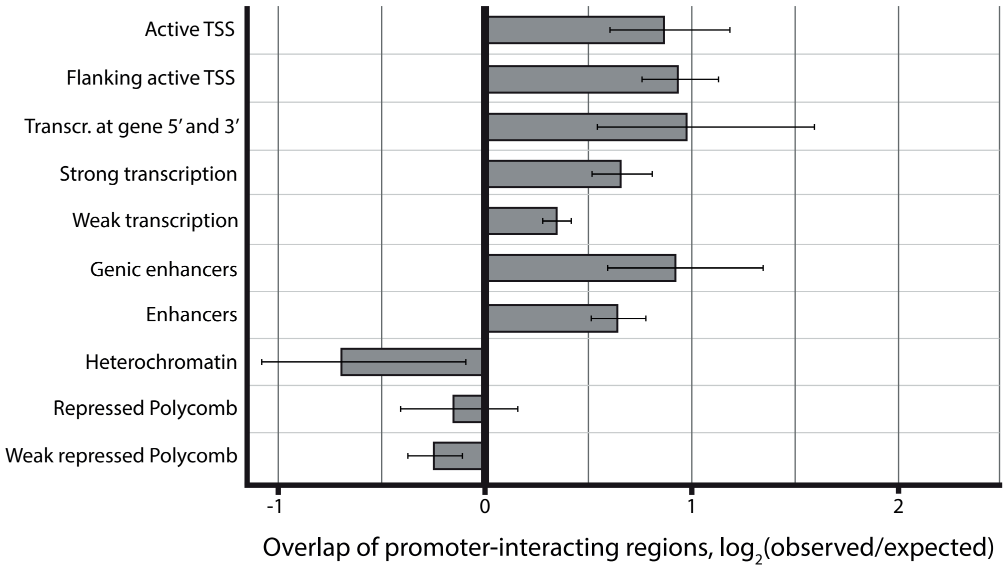

Figure S5. Enrichment of various ChromHMM chromatin types within detected promoter interacting regions (PIRs). ChromHMM chromatin state annotation for HeLa cells was downloaded from Roadmap Epigenomics portal (see methods). Rare chromatin states were excluded from analysis. Enrichment is represented as a log_2 ratio between the observed number of PIRs inside specific chromatin type and expected number of such PIRs. The latter is computed as average overlap between random distance-matched set of fragments and corresponding functional annotation. Error bars show s.d. of overlaps across 100 such random sets.

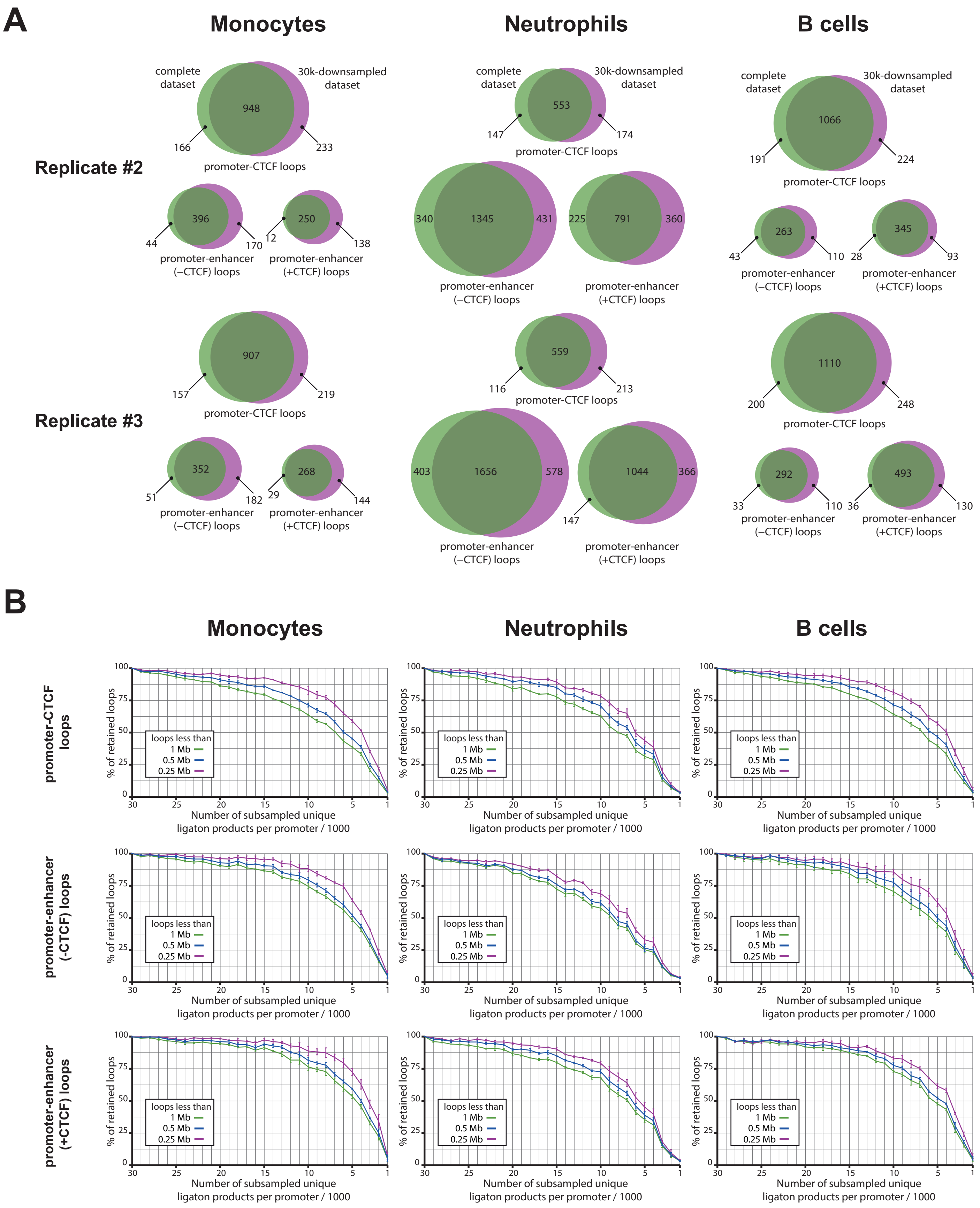

Figure S6. Additional results on retention of functional loops detected during promoter Capture-C data downsampling. A. Venn diagrams, which show CHiCAGO-detected functional loops pertaining to 1,000 randomly chosen promoters, in complete datasets (green circles, median numbers of ligation products per promoter are 50,251 for monocytes, 64,827 for neutrophils and 63,743 for B cells) and subsampled datasets (magenta circles), containing exactly 30,000 ligation products per promoter. Diagrams show data on the remaining six experiments not shown in Fig. 5. Note substantial number of apparent false positive loops arising in the process of subsampling. B. Proportion of CHiCAGO-detectable loops retained in datasets subsampled to different levels of per promoter coverage. Loops conservatively detected in both complete and 30k-downsampled datasets were considered to comprise the standard pool of interactions (100%). Loops in different size ranges are depicted in different colours. Averages of three independent downsampling experiments are shown, error bars represent s.d.

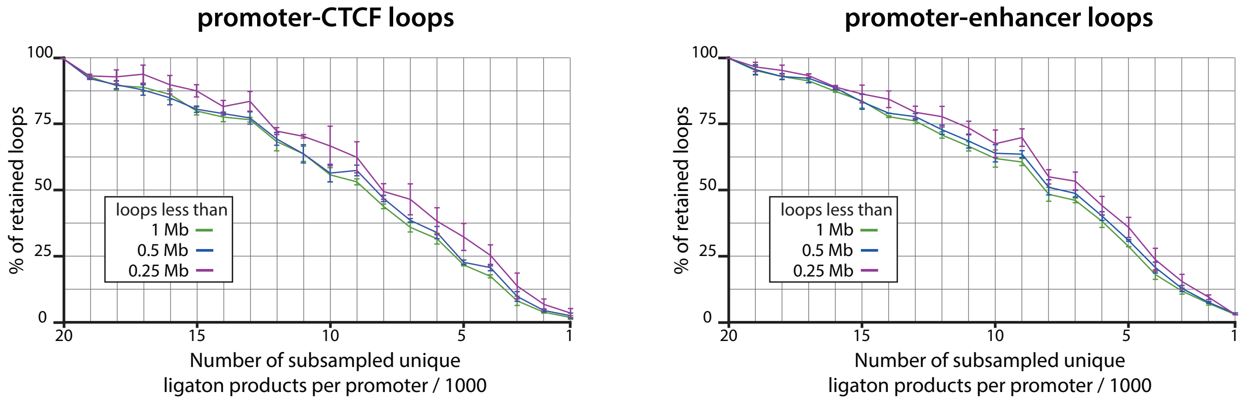

Figure S7. Retention of functional loops being detected during downsampling of promoter Capture-C data generated in Chesi et al.[38] Only 100 best covered promoters were used for the analysis. Loops conservatively detected in both complete and 20k-downsampled datasets were considered to comprise the standard pool of interactions (100%). Loops in different size ranges are depicted in different colours. Averages of three independent downsampling experiments are shown, error bars represent s.d.
